# The Tooth and Skin Colour Interrelationship across the Different Ethnic Groups

**DOI:** 10.1155/2014/146028

**Published:** 2014-07-01

**Authors:** Satheesh B. Haralur, Ahmed Mohammed Dibas, Nabil Abdullah Almelhi, Dhafer Ali Al-Qahtani

**Affiliations:** ^1^Department of Prosthodontics, College of Dentistry, King Khalid University, P.O. Box 3263, Abha 61471, Saudi Arabia; ^2^College of Dentistry, King Khalid University, P.O. Box 3263, Abha 61471, Saudi Arabia

## Abstract

*Objectives*. The purpose of the study was to investigate the relation between skin and tooth colour parameters in various ethnic groups. *Materials and Methods*. Saudi Arabian, Indian, African, and East Asian ethnic groups of 75 each were included in the study. The tooth colour was determined by spectrophotometer in CIELAB parameters. The skin colour was measured at earlobe, forehead, and malar locations by clinical skin photography. The data was statistically analysed by one-way ANOVA and correlation tests. *Results*. The “*L*” vale for the Saudi Arabian group had a strong correlation at earlobe location (*r* = 0.275), while correlation was found at forehead (*r* = 0.271) and malar region (*r* = 0.261) with Indian ethnic group. A strong negative correlation was observed in African ethnic group at all three locations for “*L*” parameter. The redness value “*a*” is found to have strong negative linear correlation between the earlobe and tooth for Saudi Arabian (*r* = −0.240) and Indian ethnic groups (*r* = −0.268). The “*b*” showed no correlation with skin location in all groups except positive correlation in African ethnic groups. *Conclusions*. The strong correlation was found between the skin and tooth colour parameters; hence the skin colour can be used as a guide for artificial tooth selection in edentulous patients.

## 1. Introduction

The restoration of patient's aesthetic and functional requirements are the main pillars of prosthetic rehabilitation [1]. The critical component of dental aesthetics is to reestablish the smile by teeth with proper arrangement in harmony with surrounding soft tissues and face [2]. The selected teeth in the prosthesis are desired to replicate the natural teeth in its surface form, translucency, and colour [3]. The tooth colour is influenced by both intrinsic and extrinsic factors [4]. The light absorption and scattering properties of enamel and dentin are intrinsic influencing factors. These intrinsic properties are affected by multiple factors like dentinogenesis imperfecta, tetracycline staining, teeth vitality, age, and dental caries [5, 6]. The extrinsic factors are external stains formed due to the combined effect of diet, restoration, and smoking [6, 7]. The colour is the psychophysical outcome of both optical properties of teeth and the observer [8]. Gender, ethnicity, and age also affect the tooth colour [9, 10]. The teeth are darker in older people in comparison to the younger population; it is attributed to continuous secondary dentin deposition. Women have lighter teeth than their men counterpart.

The selection of tooth with a proper shade has been shown to positively influence the patient's aesthetic perception and improved prosthesis acceptance [11]. The developing countries have large edentulous population due to the highly prevalent periodontal diseases [12, 13]. A sizable number of the people in these countries are in need of complete denture rehabilitation at early adult age. The properly fabricated complete denture prosthesis enables these patients to lead a normal social life. The remaining teeth are a crucial reference for selection of tooth shade during complete or partial denture fabrication [14]. The lack of this reference makes shade selection procedure a challenging and subjective exercise. Other factors suggested as guidelines in the dental literature include age, sex, and colour of skin, hair, and eye [15, 16]. The eye colour as a guideline is disregarded by many due to its size and distance away from teeth. The hair is not a reliable guide due to its rapid change in colour compared to the teeth and frequent change of colour by the patient [17]. Hence most researchers consider the face skin colour as a more predictable reference for artificial tooth selection during complete denture fabrication. Majority of the dental researchers advocate the people with darker skin complexions to have corresponding darker teeth while fair complexion individuals with lighter teeth. According to the researchers, this correlation makes the teeth colour harmonise with corresponding face skin tone in the background. Few researchers have also shown the inverse relation of tooth with skin colour [18]. Some authors dismissed the existence of any correlation between facial skin and tooth colour. General concession is to give significant weight for patient's perception while selecting the teeth colour in edentulous patients. The patient choice is predominantly influenced by many social and psychological factors. The patients tend to select white teeth in developing countries. The existing studies are few and contradict in their observation on colour correlation between teeth and skin. Majority of the researches conducted are limited to one ethnic group so the results may not be applicable to other racial groups. Thus, it is desirable to evaluate this correlation in the interracial groups with the larger variation of skin colour to have a better understanding of this correlation. Hence the study included the multiple races comprising East Asian, Indian, Saudi Arabian, and African ethnic groups. The objective of the study was to evaluate the relation between tooth colour and skin complexion in Arabic, Indian, African, and East Asian populations. The finding of the study will help in furthering the knowledge on tooth and skin complexion correlation. The study outcome will help the clinician in selecting teeth in harmony with patient's skin complexion.

## 2. Material and Methods

### 2.1. Sample Size and Selection

The College of Dentistry, King Khalid University, is situated in the southern part of Saudi Arabia. It is one of the largest dental institutes in the country. The King Khalid University dental clinics offer the free dental service to the needy public including Saudi and other nationals.

The institute's ethical committee approval was obtained for the study. The study was conducted at College of Dentistry, King Khalid University, in the first semester of 2014. The sample population was comprised of the individuals attending dental clinics seeking the dental treatment. The present cross-sectional survey was performed on stratified random samples of different nationalities. The ethnic groups evaluated were the East Asian (Philippines, Indonesia), Indian region (India, Pakistan, Bangladesh), Saudi Arabian, and African continent nationals. The sampled population for each ethnic group comprised 75 individuals. The Group 1 was of Saudi Arabian nationals; Group 2 was for Indian region, Group 3 was from African continent, and Group involved East Asian ethnic groups. The volunteers involved in the study were within the age range of 20 to 50 years. In the beginning, the first author trained the examiners involved in the study on proper medical digital photography and tooth shade selection procedure. The study inclusion criteria were the presence of the fully erupted, noncarious, unrestored maxillary central incisors. The tooth with direct restorations, veneer, and crown was excluded from the study. The additional excluding criteria were root canal treatment, tooth bleaching, intrinsic staining, and extrinsic staining due to smoking or tobacco chewing. The patients included in the study were devoid of any skin diseases, postsurgical cicatrices, or malformations of the face and skin bleaching. The excessive skin tanning, postradiation therapy, and patients not willing to participate were also excluded from the study.

### 2.2. Skin Colour Determination

In the present study, the facial skin colour was determined by the skin surface photography. The previous researcher's recommendations [19, 20] were followed during the skin surface photography. The variables like light source, camera, exposure, focal length, white balance, and patient positioning were duly considered, and care was taken to prevent their influence during photography.

The NIKON digital single-lens-reflex (DSLR) camera with the resolution of 15 megapixels was used in the study. The volunteers were requested to wash the face with a mild face wash and pat dry the face to remove the presence of dirt or cosmetics. They were made to wait for 15 minutes in a temperature-controlled room (23 ± 2 degree) after the face wash. Camera setting was standardized for the dermatological photography with the focal length at 90 to 120 mm macrolens, shutter speed of 1/250 second, and an aperture of f/16. The nonreflective light blue cloth was used as background during photography. The due care was followed for light source standardization for a better quality image and to prevent skin colour distortion. The two light sources with diffusers were used as a light source. They were positioned at 45 degree angle to the patient in the front, one light from above in a sagittal plane perpendicular to the frontal one aiming downward. The patient was seated on a chair three feet from the background; camera lenses axis was maintained at patient's eye level. The photographic distractors like hats, jewellery, and eyeglasses were removed before the photograph. The frontal view photographs were made for the study. It was standardized by using a Frankfort horizontal plane as the anatomic reference plane. An imaginary line from the superior part of tragus to infraorbital line is kept parallel to the floor, and midsagittal plane in the viewfinder is used for upright position of head. The Adobe Photoshop (Adobe Systems Incorporated, San Jose, CA, USA) was used to determine the CIELAB value of the skin at three different landmarks in the face. The landmarks used for the study were forehead 5 mm above the nasal bridge, right earlobe, and left malar area at junction of ala-tragus line and vertical line from outer canthus [21]. The facial skin colour was recorded at three separate locations to understand the colour correlation at different skin location. It was aimed at eliminating the aberration due to multiple factors like sunburn and mild skin inflammations.

### 2.3. Tooth Colour Determination

The tooth colour was measured on both maxillary central incisors at the middle third of the labial portion. The dental prophylaxis was performed on target teeth along with pumice and water mixture polishing prior to the shade selection. The tooth shade selection was made by utilizing Vita Easy shade spectrophotometer (VITA Zahnfabrik GmbH, Bad Säckingen, Germany). The instrument has built-in corrected light source from fiber-optic light at its probe. Hence the instrument can record tooth shades under any light condition.

The Vita Easy shade consists of a base and hand piece. The calibration of the instrument was done by placing a hand piece over the provided calibration ceramic disk. The white light emitted from small probe tip is utilized to illuminate the tooth and reflected light from the tooth is received to identify the colour.

The single tooth option was selected from the shade selection menu. The probe tip was held perpendicular to tooth flushing the whole surface. The measurement button is pressed to initiate the process and is held in a stable manner until the long beep. The long beep indicates the completion of the measurement. A similar procedure is followed to measure the shade from both maxillary incisors. The average tooth colour from both incisors is considered as the tooth colour for the individual.

### 2.4. Statistical Analysis

The obtained data were analysed by one-way ANOVA to evaluate the possible difference in means between the different groups. The strength of the relation between the skin and tooth colour was identified by correlation coefficients. The SPSS 18 (IBM Co., USA) program was used for analysis of statistical data.

## 3. Results

Table 1 shows the mean CIELAB values for teeth and facial skin in three locations across the different racial groups. The teeth mean “*L*” value for Saudi nationals was 79.26 (3.16), 80.59 (3.23) for Asians, 78.95 (4.33) for African nationals, and 78.33 (2.10) for East Asian population. The tooth mean “*a*” and “*b*” values for same populations were 0.85 (0.99), 21.02 (3.84) for Saudi Arabian; 0.54 (0.95), 19.57 (4.01) for Asian; 1.132 (1.51), 19.30 (4.95) for African; and 0.95 (0.56), 17.52 (2.75) for East Asian ethnic groups.

The one-way ANOVA analysis (Table 2) to find the possible difference in mean colour values across the ethnic groups showed statistically significant difference in all parameters. The *P* value in respect to parameter “*L*” was 0.009 and 0.020 for “*a*” and parameter *b* had 0.003. As expected, the facial colour values in all locations between different nationalities showed the statistically significant difference. The *P* value for all colour parameters including *L*, *a*, and *b* was 0.000 for all facial skin locations.

Moderate to strong linear relationship between tooth and facial skin colour was revealed by the correlation analysis (Table 3). The strength of correlation was different between groups. Group 1 “*L*” parameter correlated significantly with earlobe skin (*r* = 0.275, *P* = 0.018), while no significant correlation in other skin location. For the “*a*” factor the tooth colour was found to have strong negative correlation with skin colour in ear lobe location with *P* = 0.04 and *r* = −0.240. Group 1 showed no significant relation in “*b*” parameter with skin colour across all the locations.

The tooth colour “*L*” value in Group 2 showed a strong positive correlation in malar and forehead skin colour. The “*r*” factor for malar and forehead was 0.271 and 0.261, respectively, with *P* value for each group being 0.024 and 0.019. The “*a*” value of tooth colour had the same trend shown in Group 1, and it had a significant correlation (*P* = 0.020) only with skin colour in ear lobe location. As observed in Group 1, the “*b*” value showed no significant correlation with any facial skin location. Group 3 had a different correlation compared to Group 1 and Group 2. The tooth “*L*” value was negatively correlated with skin colour in all locations. The earlobe, malar, and forehead had −0.399, −0.415, and −0.390 “*r*” factors, respectively. The “*a*” and “*b*” values in tooth for Group 3 had a strong positive linear correlation (*P* < 0.05) across all skin locations. The teeth and skin colour were not significantly correlated for any colour parameters for Group 4 (*P* > 0.05).

## 4. Discussion

The colour is a psychophysical response of an individual toward the light interaction with the object. The physical properties of light and hard tissues affect the colour. The perceived colour is also influenced by psychological precondition of an individual [22]. The tooth colour complimenting the skin colour is essential for the aesthetically successful facial restorations. Lack of pre extraction records and remaining teeth makes the tooth shade selection in complete denture patient entirely subjective. The tooth shade selection will be more objective if another facial appearance feature is used as a reliable guide. It will help the artificial dentures to harmonize better with the facial appearance, and patient compliance will be improved. The spectrophotometer was utilized to find the tooth colour in the present study. It is more consistent and objective than the manual or colorimeter measurement [23]. The spectrophotometer colour measurement eliminates the impact of different light sources and interexaminer bias. The skin surface photography was employed to determine the skin colour in the present study. The regulated light source, camera setting, and lenses have been suggested by researchers for the clinical facial photography. The results of the study support the research hypothesis of finding a significant correlation between skin and tooth colour. The data showed a linear correlation between the tooth and skin colour among all ethnic groups evaluated in the study. The correlation strength and linear correlation were varied across the ethnic groups. The tooth lightness value was significantly (*P* = 0.09) different between the groups. It indicates the inherent difference in the tooth “*L*” value between different ethnic groups. The “*L*” value between the tooth and skin was significantly correlated in Group 1 at ear lobe area (*r* = 0.217) while Group 2 had a positive linear correlation at forehead location (*r* = 0.271) and malar region (*r* = 0.261). Group 4 also had the tooth *L* value significant correlation with forehead skin colour (*r* = 0.042). The result is in agreement with finding of Lagouvardos et al. [21]; they found the skin lightness of malar and earlobe area was positively correlated with the lightness of teeth. The present study revealed the African ethnic group (Group 3) had a strong negative relationship between the lightness of the tooth with lightness of skin in all three locations. The earlobe, forehead, and malar locations had “*r*” value of −0.399, −0.415, and −0.390, respectively. This observation is in agreement with the finding of the previous studies on black African population [18]. The redness “*a*” value between the teeth and skin at ear lobe is inversely proportional in Group 1 (*r* = −0.240) and Group 2 (*r* = −0.268). Group 3 had contradictory positive correlation with skin at all three locations. The results of the study were in confirmation with the finding of a study by Jahangiri et al. [24], who found the similar negative relationship between teeth and skin colour. The yellow “*b*” value of the tooth showed no correlation with skin in all groups except Group 3, which showed the positive correlation in all three face locations. The earlier researchers like Dummett et al. [25], Esan et al. [26], Gazalo-Diaz et al. [27], and Hassel et al. [28] reported no relationship between skin and tooth colour. The justification for the differences in the results may be attributed to the difference in methodology. Most of the previous studies used the manual method for tooth shade selection. The compact makeup shades determined the skin colour; it was broadly categorized compared to identifying individual colour. Hence the human bias in manual tooth shade selection and limited skin colour range could have influenced the study outcomes. The results of the study indicate the difference in correlation between tooth and skin colour values in different populations. The finding of the study reveals the lightness of the tooth and skin colour is directly proportional in all examined ethnic groups except the African group. The redness of the teeth is inversely proportional to the skin colour in the majority population. The correlation between skin and tooth colour is not universal across all ethnic groups. The clinicians should understand the type of correlation before artificial tooth selection. The finding of the study helps the dentist to understand the difference of tooth and skin colour association in various ethnic groups and assist the clinician in selecting the artificial teeth in harmony with skin colour. It will also help in the selection of the skin colour while fabricating maxillofacial prosthesis.

Limitation of the study includes the tooth colour was determined from central incisors. The further exploration of the skin colour relation with more yellow and red teeth like canine and premolar is required. The spectrometer with its flat probe tip may result in edge loss effect during colour selection. Further research is needed to understand the role of age on the tooth and skin colour parameters and its subsequent effect on their correlation.

## 5. Conclusion

Within the limitation of the study, a significant correlation was found between tooth and skin color. The Saudi Arabian, East Asian, and Indian groups had positive linear correlation with the lightness value between tooth and skin color. The correlation was observed with skin “*L*” parameter in all three facial locations. The redness “*a*” value for tooth and facial skin showed the negative linear association in the same population. The association in the African ethnic groups showed the negative correlation between “*L*” value and positive correlation with “*a*” value. The results indicate the skin colour can be used as a reliable guide for the artificial tooth colour selection in the absence of natural teeth. The outcome is helpful in understanding the appropriate skin colour for maxillofacial prosthesis.

## Figures and Tables

**Table 1:** Mean *L*, *a*, and *b* values and standard deviation among all ethnic groups.

Group	Location	*L* value		*a* Value		*b* value	
		Mean	SD	Mean	SD	Mean	SD
Group 1	Tooth	79.26	3.16	0.853	0.99	21.05	3.84
	Ear lobe	44.36	7.06	27.46	4.50	22.12	3.9
	Forehead	51.77	6.17	22.95	3.25	25.36	3.44
	Malar	50.96	6.55	28.78	3.19	24.32	2.96

Group 2	Tooth	80.59	3.23	0.546	0.952	19.57	4.01
	Ear lobe	40.97	9.27	29.09	5.23	22.05	4.80
	Forehead	45.11	7.34	29.72	5.13	24.23	3.77
	Malar	44.84	7.08	30.81	5.41	23.85	3.31

Group 3	Tooth	78.95	4.33	1.132	1.513	19.30	4.95
	Ear lobe	37.79	14.82	26.55	7.96	19.32	6.91
	Forehead	40.61	13.20	26.91	7.56	20.33	6.22
	Malar	41.15	13.58	28.00	7.55	20.23	5.53

Group 4	Tooth	78.33	2.10	0.955	0.564	17.52	2.75
	Ear lobe	190.18	8.44	127	9.37	116.32	10.07
	Forehead	195.95	8.86	130.05	12.23	119.00	13.82
	Malar	192.73	23.25	129.27	11.78	119.09	12.61

**Table 2:** One-way ANOVA analysis for tooth *L*, *a*, and *b* values.

	Sum of squares	df	Mean square	*F*	Significance
T_*L*_					
Between groups	145.265	3	48.422	3.917	0.009
Within groups	2991.633	287	12.362		
Total	**3136.898**	**300**			

T_*a*_					
Between groups	13.134	3	4.378	3.352	0.020
Within groups	316.098	287			
Total	**329.232**	**300**			

T_*b*_					
Between groups	252.172	3	84.057	4.785	0.003
Within groups	4503.026	287	17.566		
Total	**4250.854**	**300**			

**Table 3:** Pearson's correlation coefficient matrix of teeth and skin colour at different locations.

Location	Values	Group 1	Group 2	Group 3	Group 4
Earlobe	*L*	0.275∗	0.192	−0.399∗∗	−0.275
	*a*	−0.240∗	−0.268∗	0.348∗∗	0.037
	*b*	0.029	−0.142	0.479∗∗	−0.341

Forehead	*L*	0.217	0.271∗	−0.415∗∗	0.041∗
	*a*	0.038	−0.164	0.285∗∗	0.024
	*b*	−0.022	−0.065	0.502∗∗	−0.351

Malar	*L*	0.186	0.261∗	−0.390∗∗	−0.193
	*a*	0.087	−0.185	0.447∗∗	0.083
	*b*	0.061	−0.094	0.483∗∗	−0.354

∗Significant at *P* = 0.05. ∗∗Significant at *P* = 0.01.
